# An inhibitory effect on the nuclear accumulation of phospho-STAT1 by its unphosphorylated form

**DOI:** 10.1186/s12964-022-00841-3

**Published:** 2022-03-31

**Authors:** Priyanka Rajeev Menon, Julia Staab, Anke Gregus, Oliver Wirths, Thomas Meyer

**Affiliations:** 1 Department of Psychosomatic Medicine and Psychotherapy, University Medical Centre Göttingen, and German Centre for Cardiovascular Research (DZHK), Partner Site Göttingen, Göttingen, Germany; 2 Department of Psychiatry and Psychotherapy, University Medical Centre Göttingen, Göttingen, Germany

**Keywords:** STAT1, JAK-STAT signalling, Dimerization, Nuclear accumulation, Interferon-induced gene expression

## Abstract

**Background:**

Unphosphorylated signal transducer and activator of transcription 1 (U-STAT1) has been reported to elicit a distinct gene expression profile as compared to tyrosine-phosphorylated STAT1 (P-STAT1) homodimers. However, the impact of U-STAT1 on the IFNγ-induced immune response mediated by P-STAT1 is unknown. By generating a double mutant of STAT1 with mutation R602L in the Src-homology 2 (SH2) domain and Y701F in the carboxy-terminal transactivation domain mimicking U-STAT1, we investigated the effects of U-STAT1 on P-STAT1-mediated signal transduction.

**Results:**

In this study, we discovered a novel activity of U-STAT1 that alters the nucleo-cytoplasmic distribution of cytokine-stimulated P-STAT1. While the dimerization-deficient mutant R602L/Y701F was not able to display cytokine-induced nuclear accumulation, it inhibited the nuclear accumulation of co-expressed IFNγ-stimulated wild-type P-STAT1. Disruption of the anti-parallel dimer interface in the R602L/Y701F mutant via additional R274W and T385A mutations did not rescue the impaired nuclear accumulation of co-expressed P-STAT1. The mutant U-STAT1 affected neither the binding of co-expressed P-STAT1 to gamma-activated sites in vitro, nor the transcription of reporter constructs and the activation of STAT1 target genes. However, the nuclear accumulation of P-STAT1 was diminished in the presence of mutant U-STAT1, which was not restored by mutations reducing the DNA affinity of mutant U-STAT1. Whereas single mutations in the amino-terminus of dimerization-deficient U-STAT1 similarly inhibited the nuclear accumulation of co-expressed P-STAT1, a complete deletion of the amino-terminus restored cytokine-stimulated nuclear accumulation of P-STAT1. Likewise, the disruption of a dimer-specific nuclear localization signal also rescued the U-STAT1-mediated inhibition of P-STAT1 nuclear accumulation.

**Conclusion:**

Our data demonstrate a novel role of U-STAT1 in affecting nuclear accumulation of P-STAT1, such that a high intracellular concentration of U-STAT1 inhibits the detection of nuclear P-STAT1 in immunofluorescence assays. These observations hint at a possible physiological function of U-STAT1 in buffering the nuclear import of P-STAT1, while preserving IFNγ-induced gene expression. Based on these results, we propose a model of a hypothetical import structure, the assembly of which is impaired under high concentrations of U-STAT1. This mechanism maintains high levels of cytoplasmic STAT1, while simultaneously retaining signal transduction by IFNγ.

**Video Abstract**

**Supplementary Information:**

The online version contains supplementary material available at 10.1186/s12964-022-00841-3.

## Background

The structure and function of signal transducers and activators of transcription (STATs) have been extensively studied, leading to the establishment of a signal transduction pathway employed by several cytokines to induce cellular responses driving inflammation, differentiation, proliferation and apoptosis. STATs, which together with their cytokine-receptor-associated Janus kinases (JAKs) form the JAK-STAT pathway, become phosphorylated by JAKs at a single tyrosine residue upon cytokine binding to cell-surface receptors and translocate to the nucleus as dimers to initiate cytokine-induced gene transcription. The founding member of the protein family of STATs, the interferon-dependent transcription factor STAT1, mediates anti-viral immune responses induced by type I and type II interferons (IFNs) [1–4].

Crystallographic studies of STAT1 have elucidated its modular structure, comprised of six domains, namely (1) an N-terminal domain, (2) a coiled-coil domain, (3) a DNA-binding domain, (4) a linker domain, (5) an Src-homology 2 (SH2) domain, and (6) a C-terminal transactivation domain [5, 6]. STAT1 is localized predominantly in the cytoplasm prior to cytokine stimulation and upon receptor activation by IFNγ, the receptor-associated JAKs phosphorylate a critical tyrosine residue at position 701 in the STAT1 transactivation domain producing phosphorylated STAT1 (P-STAT1). STAT1 is known to dimerize in two possible orientations: (i) as a parallel dimer stabilized by reciprocal interactions between the phospho-tyrosine Y701 and the R602 residue in the SH2 domain or (ii) as an anti-parallel dimer formed by associations between the coiled-coil domain of a protomer and the DNA-binding domain of its partner, and vice-versa [5–8]. While the unphosphorylated protein (U-STAT1) can exist as monomers or anti-parallel dimers, tyrosine phosphorylation shifts the dimeric conformation from the predominant anti-parallel orientation to parallel dimers [9]. This shift can occur either by constitutive dissociation-association between monomers or around a tether formed by homotypic interactions between the amino-terminal regions of the two monomers [10]. The switching of dimer conformation upon tyrosine phosphorylation is also imperative to expose a nuclear localization signal (NLS) formed out of the STAT1 DNA-binding domains of both parallel oriented protomers, which is recognized by members of the importin α family to transport P-STAT1 to the nucleus [11, 12]. As a consequence, U-STAT1 molecules, which exist as monomers or anti-parallel dimers, are excluded from importin-dependent nuclear translocation. While STAT1 proteins constitutively shuttle between the cytosol and nucleus via diffusion and associations with nuclear pore complexes, a cytokine-induced nuclear accumulation of P-STAT1 to initiate gene transcription requires importin-mediated transport [13–15].

IFN-induced recruitment of P-STAT1 to the promoter of the *STAT1* gene up-regulates its own expression in a positive feedback loop, resulting in high concentrations of U-STAT1. Thus, STAT1 is its own target gene, and due to this phenomenon, U-STAT1 has been reported to prolong interferon responses after initial activation [16, 17]. In addition, STAT1 is required for caspase expression and tumor necrosis factor α (TNFα)-induced apoptosis, which is lost in STAT1-deficient U3A cells [18]. However, reconstitution of these cells with U-STAT1 restores sensitivity to apoptosis, indicating that STAT1 has regulatory functions independent of tyrosine phosphorylation. Although U-STAT1 acts as a mediator for apoptotic cell death, it is unclear whether there are any interactions between U-STAT1 and P-STAT1 affecting the equilibrium between canonical and non-canonical functions of STAT1.

Here, we investigate the effects of a high concentration of U-STAT1 on P-STAT1-mediated signal transduction. By introducing a substitution of the critical tyrosine residue at 701 to phenylalanine and an arginine-to-leucine exchange at 602, we have generated a double mutant construct mimicking U-STAT1 that cannot form dimers with wild-type (WT) STAT1 in the parallel orientation. To further disrupt anti-parallel interactions in this double mutant, we have additionally mutated residues in the coiled-coil domain and DNA-binding domain to render a complete dimerization-deficient mutant U-STAT1. Using these two constructs, we attempt to study the effect of U-STAT1 expressed in high concentrations on the intracellular activities of P-STAT1. Our data provide a novel outlook on the interplay between tyrosine-phosphorylated and unphosphorylated STAT1 molecules in the regulation of IFNγ-induced STAT1 signaling at the level of nuclear import.

## Materials and methods

### Plasmids, mutagenesis and cell culture

The following STAT1 expression vectors were used for this study: pEGFPN1-STAT1α (WT-GFP [19]), pEGFPN1-STAT1α-ΔN (ΔN-GFP; a kind gift from Prof. Uwe Vinkemeier, University of Nottingham), pSTAT1α-Flag (WT-Flag), and pcDNA3.1-STAT1α [14]. WT-GFP coding for a carboxy-terminal fusion of full-length human STAT1 (amino acids 1–746) with green fluorescent protein (GFP) was used as a template to introduce point mutations by site-directed mutagenesis using the QuikChange II kit from Stratagene, according to the manufacturer’s instructions. The following primers were used with mutated codons underlined (only forward primers are shown):

- R274Wf; 5′-GAG AGT CTG CAG CAA GTT TGG CAG CAG CTT AAA AAG TTG-3′,
- T385Af; 5′-GAA GTT CAA CAT TTT GGG CGC GCA CAC AAA AGT GAT GAA C-3′,
- L407A/L409Af; 5′-GGC TGA ATT TCG GCA CGC GCA AGC GAA AGA ACA GAA AAA TGC -3′,
- V426D/T427Df; 5′-GAG GGT CCT CTC ATC GAT GAT GAA GAG CTT CAC TC-3′,
- E524Af; 5′-CTG AAC ATG TTG GGA GCG AAG CTT CTT GGT CCT AAC GCC -3′,
- R586Ef; 5′-GGC TTC ATC AGC AAG GAG GAA GAG CGT GCC CTG TTG -3′,
- R602Lf; 5′-CCG GGG ACC TTC CTG CTG CTG TTC AGT GAG AGC TCC-3′ and
- Y701Ff; 5′-CCT AAA GGA ACT GGA TTT ATC AAG ACT GAG TTG-3′.

All mutations were verified by standard dideoxy-termination DNA sequencing (Seqlab) and the resulting plasmids were used for transfection. HeLa cells and STAT1-negative U3A cells [20] were cultured in a humidified 5% CO_2_ atmosphere at 37 °C in either Roswell Park Memorial Institute (RPMI) 1640 medium supplemented with 10% foetal bovine serum (FBS) (Biochrom), 100 IU/ml penicillin/streptomycin (for HeLa cells) or Dulbecco’s modified Eagle’s medium (DMEM) (PAA Laboratories) supplemented with 10% FBS, 100 IU/ml penicillin/ streptomycin and 0.04 μg/ml puromycin (Sigma-Aldrich) (for U3A cells). Cells were transfected with MegaTran2.0 (Origene) and on the next day either left untreated or alternatively stimulated with 50 ng/ml of recombinant human interferon-γ (IFNγ) (Biomol) or with 50 ng/ml IFNα (Biomol) for the indicated times.

### Protein extraction and Western blotting

GFP-tagged or untagged STAT1-expressing cells grown on 6-well dishes were lysed on ice for 5 min in 50 µl cytoplasmic extraction buffer (20 mM HEPES, pH 7.4, 10 mM KCl, 10% (v/v) glycerol, 1 mM EDTA, 0.1 mM Na_3_VO_4_, 0.1% IGEPAL-CA-360, 3 mM 1.4-dithiothreitol (DTT), 0.4 mM Pefabloc (Sigma-Aldrich), and Complete Mini protease inhibitors (Roche)). The extracts were centrifuged at 16,000*g* for 15 s at 4 °C. The supernatants were spun again for 5 min at 16,000*g* and collected as cytoplasmic extracts. The pellets from the first centrifugation step were resuspended in 50 µl nuclear extraction buffer (20 mM HEPES, pH 7.4, 420 mM KCl, 20% (v/v) glycerol, 1 mM EDTA, 0.1 mM Na_3_VO_4_, 3 mM DTT, 0.4 mM Pefabloc, and Complete Mini protease inhibitors) and left on ice for 30 min. After centrifugation at 16,000*g* for 15 min and 4 °C, the nuclear extracts were mixed with the same volume of cytoplasmic extracts from the corresponding sample to generate whole cell lysates, whereas for subcellular fractionation, the extracts were stored separately. The cellular extracts were boiled for 3 min in sodium dodecyl sulphate (SDS) sample buffer and resolved by 10% SDS-polyacrylamide gel electrophoresis (SDS-PAGE) with subsequent transfer onto polyvinylidene difluoride (PVDF) membranes. The membranes were incubated with either a rabbit monoclonal phospho-Tyr701-specific STAT1 antibody (Cell Signaling Technology, 58D6) or monoclonal pan-STAT1 antibody (Cell Signaling Technology, D1K9Y) overnight. To investigate the expression and phosphorylation of STAT3, monoclonal phospho-Tyr705-specific STAT3 (Cell Signaling, D3A7) and monoclonal pan-STAT3 antibodies (Cell Signalling, D1B2J) were used. For fractionation assays, GAPDH Rabbit mAb (14C10, Cell Signaling Technology) and lamin A (H-102, Santa Cruz Biotechnology) were used in addition. After extensive washing, the blots were exposed to a conjugated secondary anti-rabbit IRDye 800CW antibody (LI-COR). Immunoreactivity was detected using a LI-COR Odyssey imaging system.

### In vitro dephosphorylation and phosphorylation assays

The in vitro dephosphorylation assay was performed at 30 °C for the indicated times using 10 µl of whole cell extracts from IFNγ-treated U3A cells expressing untagged WT in a combination with either GFP-tagged WT or mutant STAT1. The same volume of phosphatase buffer was added to the reactions (25 mM Tris-HCl, pH 7.5, 50 mM KCl, 5 mM EDTA, 20 mM DTT, 0.5 mg/ml bovine serum albumin, 2 U of the T-cell protein tyrosine phosphatase Tc45 (Biomol), and Complete protease inhibitors). Reactions were stopped by adding SDS sample buffer and boiling the samples for 3 min. Tyrosine-phosphorylated and total STAT1 were probed on different PVDF membranes by means of Western blotting. For in vitro phosphorylation assays, extracts from unstimulated U3A cells expressing untagged WT in a combination with either GFP-tagged WT or mutant STAT1 were used. The kinase buffer contained 50 mM HEPES, pH 7.4, 3 mM MgCl_2_, 3 mM MnCl_2_, 3 µM Na_3_VO_4_, 10 mM DTT, 0.1 mM ATP, and 4 μg/ml of recombinant JAK2 (Enzo Life Science). The in vitro phosphorylation reactions were carried out at 30 °C with 10 µl of whole cell extracts for the indicated times.

### Fluorescence microscopy

The kinetics of IFNγ-induced nuclear accumulation of WT and mutant STAT1 were monitored in HeLa and U3A cells by a combination of direct and indirect fluorescence microscopy. Transiently transfected cells expressing GFP-tagged/Flag-tagged and/or endogenous STAT1 in 8-well chamber slides were treated with cytokines, as indicated. After stimulation, all cells were fixed with methanol for 15 min at − 20 °C before they were permeabilized with 1% Triton X-100 in phosphate-buffered saline (PBS) for 20 min at room temperature (RT). To saturate unspecific binding sites, the cells were treated with 25% FBS-PBS (25% FBS in PBS) for 45 min while being shaken. This was followed by a 45-min incubation at RT while being shaken with the primary antibody (Monoclonal anti-Flag mouse mAb, Sigma Aldrich or Phospho-Stat1 (Tyr701) (58D6) Rabbit mAb, Cell Signaling Technology), as indicated (1: 1000 in 25% FBS-PBS). The cells were then washed thrice with PBS. For the detection of the primary antibody, a Cy3-coupled anti-rabbit IgG secondary antibody from goat (anti-mouse and anti-rabbit, Jackson Immunoresearch Laboratories, USA) (1:1000 in 25% FBS-PBS) was added and incubated for 45 min at RT while being shaken. Subsequently, nuclei were stained for 10 min with 5 μg/ml of Hoechst dye 33258 (Sigma-Aldrich). Samples were mounted in fluorescence mounting medium (Southern Biotech) and the intracellular fluorescence localization visualized using a Nikon Eclipse Ti fluorescence microscope equipped with appropriate filters. Images obtained from a Nikon DS-Qi2 camera were further processed with the NIS elements (Nikon) software. Confocal images were taken with a Nikon C2+ confocal microscope equipped with 405 nm, 488 nm and 561 nm lasers (Nikon). Fluorescence intensities were determined both in the nucleus and cytoplasm using ImageJ (NIH), and the mean nuclear-to-total-cellular fluorescence intensities including their standard deviations were calculated from 20 randomly selected transfected cells.

### Electrophoretic mobility shift assay

STAT1 variants were probed for their DNA-binding activity to duplex oligonucleotides containing a single GAS site or two tandem GAS sites by means of electrophoretic mobility shift assays (EMSA). Per reaction, 4 µl of cellular extracts from unstimulated and IFNγ-stimulated cells expressing recombinant STAT1 in the presence (HeLa cells) or absence of endogenous STAT1 (U3A cells) were incubated with 8 µl of EMSA reaction buffer containing 1 ng of the [^33^P]-labelled duplex oligonucleotide probe, generated by an end-filling reaction using the Klenow fragment (New England Biolabs). The M67 duplex oligonucleotide containing a single GAS site or the 2xGAS duplex oligonucleotide containing two GAS sites in tandem orientation were used (the GAS sites are underlined, while the respective anti-sense oligos are not shown):

- M67; 5′-TTT TCG ACA TTT CCC GTA AAT CTG-′3,
- 2xGAS; 5′-TTTTCGT TTC CCC GAA ATT GAC GGA TTT CCC CGA AAC-′3.

For competition reactions, cell lysates were incubated with [^33^P]-labelled duplex oligonucleotides in EMSA reaction buffer for 15 min at RT, and subsequently challenged by a 750-fold molar excess of unlabelled M67 DNA incubated for 10 min at RT. For super-shift assays, 20 ng of STAT1-specific antibody D1K9Y or STAT3-specific antibody D1B2J (Cell Signaling Technology) were added to the reaction for 40 min at RT. The reactions were loaded on a 4.8% 29:1 acrylamide:bisacrylamide gel at 4 °C and separated at 400 V. DNA binding was visualized on vacuum-dried gels using the laser phosphor-imaging system Typhoon FLA 9500 (GE Healthcare Life Sciences).

### Reporter gene assays

Gene induction by WT STAT1 in the presence of co-expressed mutant U-STAT1 was studied in transfected HeLa cells and reconstituted U3A cells. To this end, cells grown on 48-well plates were co-transfected in each well with the following vectors: a luciferase reporter (70 ng), a constitutively expressed β-galactosidase plasmid (200 ng), and expression plasmids encoding either GFP-tagged WT alone or in combination with other STAT1 variants (250 ng). The luciferase reporter contained a triple Ly6E STAT-binding site (termed 3xLy6E) in the IFNγ-inducible promoter region of the luciferase gene [21, 22]. One day after transfection, cells were either left untreated or treated for 6 h with IFNγ, before cell extracts were prepared with a lysis buffer containing 25 mM glycylglycine, 1% Triton X-100, 15 mM MgSO_4_, 4 mM EGTA, 0.4 mM Pefabloc, 3 mM DTT, pH 7.8, and Complete protease inhibitors. Luciferase expression was assessed with the luciferase assay system substrate solution from Promega using the luminometer Centro KS LB960 (Berthold Technologies) and normalized to the corresponding β-galactosidase activity, which was measured spectroscopically at 420 nm. The experiment was repeated in duplicate, and six independent transfections were tested for every combination of STAT1 variants and stimulation mode.

### Real-time PCR

For expression of endogenous target genes, U3A cells expressing a combination of GFP-tagged STAT1 variants as indicated were cultured for 15 h in Dulbecco’s modified Eagle’s medium supplemented with 1% FBS, before they were either left untreated or stimulated for 6 h with 50 ng/ml IFNγ. RNA was isolated using the peqGold Total RNA kit (VWR Lifesciences), and first-strand cDNA synthesis was performed using the Verso cDNA Synthesis kit (Thermo Fisher Scientific). Real-time PCR reactions were carried out in a total volume of 20 µl, containing 25 ng of cDNA, 70 nM of each specific primer, and 10 µl of Absolute Blue qPCR SYBR Green Mix (Thermo Fisher Scientific). The following primer pairs were used:

- CXCL10F; 5′-ATT CTG AGC CTA CAG CAG AG-3′,
- CXCL10R; 5′-GCT TGC AGG AAT AAT TTC AA-3′,
- GAPDHF; 5′-GAA GGT GAA GGT CGG AGT C-′3,
- GAPDHR; 5′-GAA GAT GGT GAT GGG ATT TC-′3,
- GBP1F; 5′-GGT CCA GTT GCT GAA AGA GC-3′,
- GBP1R; 5′-TGA CAG GAA GGC TCT GGT CT-3′,
- IRF1F; 5′-AGC TCA GCT GTG CGA GTG TA-3′,
- IRF1R; 5′-TAG CTG CTG TGG TCA TCA GG-3′,
- MIGF; 5′-CCA CCG AGA TCC TTA TCG AA-3′, and
- MIGR; 5′-CTA ACC GAC TTG GCT GCT TC-3′.

The PCR protocol run on an Eppendorf cycler included a denaturation step at 95 °C for 15 min, which was followed by 40 cycles of denaturation at 95 °C for 15 s, annealing at 55 °C for 30 s, and extension at 72 °C for 30 s. A melting curve analysis was performed after the final amplification step using a temperature gradient from 60 °C to 95 °C in 0.5 °C increment steps and fluorescence being measured at each temperature for a period of 10 s. All reactions were carried out in at least triplicate independent experiments. The relative expression of a transcript was normalized to the expression of *GAPDH* as determined for each sample. The ΔΔCt-method based on the formula 2^−(ΔCt target − ΔCt reference sample)^ was used to determine comparative relative expression levels.

### Data analysis

Digital images were processed by ImageJ (NIH) software and data figures were created using CorelDRAW Graphics Suite 2019. For each STAT1 variant or combination of variants and stimulation mode, means and standard deviations were calculated. Differences between WT in the presence and absence of mutant protein were assessed using Student’s *t*-tests and Mann-Whitney-Wilcoxon tests, where appropriate. Data were analyzed using the GraphPad PRISM, and a *p* value ≤ 0.05 was considered to indicate statistical significance.

## Results

### Dimerization-deficient U-STAT1 mutants inhibit cytokine-induced nuclear accumulation of the co-expressed WT protein

**Fig. 1:**
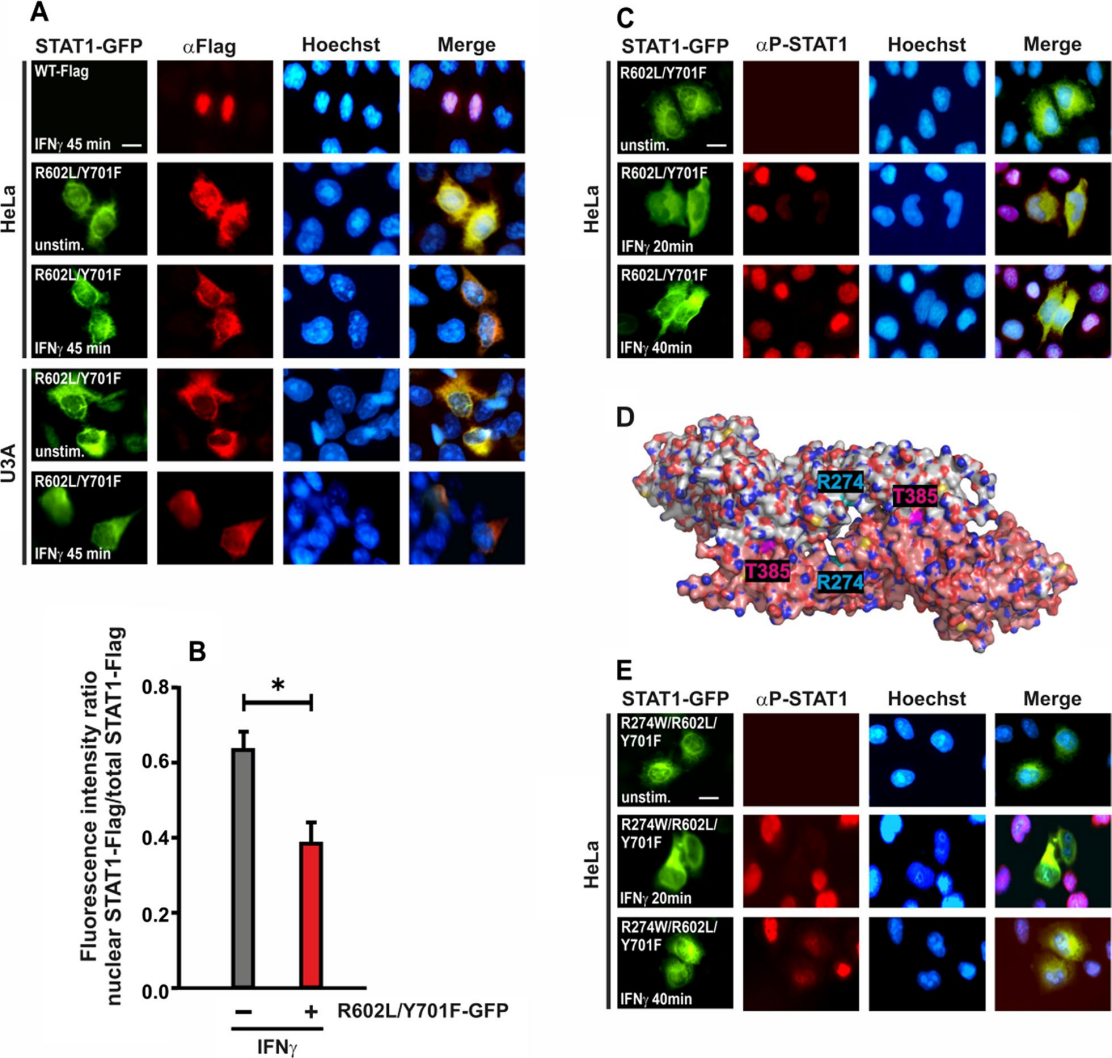
**A**, **B** Cytoplasmic retention of Flag-tagged WT STAT1 in IFNγ-stimulated cells co-expressing R602L/Y701F-GFP. Transfected STAT1-negative U3A and HeLa cells expressing R602L/Y701F-GFP and WT-Flag were treated with 50 ng/ml IFNγ for 45 min and subsequently stained with an anti-Flag antibody. **A** The fluorescence micrographs show the intracellular distribution of Flag-tagged STAT1 co-expressed in cells transfected with R602L/Y701F-GFP as well as the localization of the corresponding Hoechst-stained nuclei (n = 3 independent transfections). **B** The histogram demonstrates the nucleocytoplasmic STAT1-Flag distribution in the absence and presence of R602L/Y701F-GFP in IFNγ-treated HeLa cells, as determined by the ratio of nuclear-to-total fluorescence intensity (means ± standard deviations from n = 20 cells, *p ≤ 0.05, as assessed by a two-tailed Student’s *t* test). **C** The absence of nuclear P-STAT1 staining of the endogenous protein in HeLa cells expressing R602L/Y701F-GFP. HeLa cells were transfected with R602L/Y701F-GFP and stimulated with IFNγ for indicated times before staining with an anti-phospho-tyrosine antibody. **D** Crystal structure of the STAT1 anti-parallel dimer. A critical arginine residue at position 274 marked in cyan in the coiled-coil domains and threonine residue at position 385 marked in pink in the DNA-binding domains of the two STAT1 proteins (white and orange). Structural data were obtained from the Protein Data Bank (pdb) file 1YVL [6] for the STAT1 anti-parallel dimer. **E** The absence of nuclear P-STAT1 staining of endogenous STAT1 in HeLa cells expressing R274W/R602L/Y701F-GFP. HeLa cells were transfected with R274W/R602L/Y701F-GFP and stimulated with IFNγ for indicated times before being stained with an anti-phospho-tyrosine antibody. Scale bars in **A**, **C**, **E** mark a distance of 10 µm

The previously characterised single mutations R602L and Y701F in STAT1 lead to abolished tyrosine phosphorylation and cytokine-induced nuclear accumulation [5, 7]. Therefore, these two mutations were combined in STAT1-GFP to create a construct that mimics U-STAT1 and cannot form parallel dimers with other STAT molecules. The double mutant R602L/Y701F-GFP, when transfected as a plasmid in either HeLa or U3A cells, showed a normal expression pattern and did not accumulate in the nucleus upon IFNγ stimulation, similar to the contributing single mutants (Fig. 1A). Unexpectedly, the mutant U-STAT1 impaired cytokine-induced nuclear accumulation of the co-expressed Flag-tagged WT protein in IFNγ-stimulated HeLa and U3A cells (Fig. 1A, B). Staining with an anti-Flag antibody and a corresponding Cy3-labelled secondary antibody showed a cytoplasmic localization of Flag-tagged STAT1 in cytokine-stimulated cells co-expressing the R602L/Y701F-GFP mutant, while cells expressing only Flag-tagged STAT1 showed unaltered cytokine-induced nuclear accumulation (Fig. 1A, row 1). To determine whether this phenomenon also impacted the distribution of endogenous P-STAT1, HeLa cells were transfected with R602L/Y701F-GFP and subsequently stimulated with IFNγ for the indicated times followed by staining with an antibody against P-STAT1. This antibody exclusively detects endogenous protein and does not label the recombinant protein lacking the phospho-tyrosine 701. Surprisingly, cytokine-stimulated HeLa cells expressing R602L/Y701F-GFP showed an absence of immune-detectable tyrosine-phosphorylated STAT1 in the nucleus and cytosol, while cells not expressing mutant U-STAT1 demonstrated a clear nuclear accumulation of the endogenous P-STAT1 (Fig. 1C).

To abolish possible anti-parallel dimer interactions between mutant U-STAT1 and co-expressed WT, we sequentially introduced two additional mutations, namely R274W and T385A, in the R602L/Y701F-GFP protein (Fig. 1D). Previous data from our group demonstrated that the hyper-active R274W mutation in the coiled-coil domain is a human gain-of-function (GOF) mutation causing chronic mucocutaneous candidiasis by the disruption of the anti-parallel dimer interface [23]. As depicted in Fig. 1E, the triple mutant R274W/R602L/Y701F behaved in a similar manner as the double mutant R602L/Y701F, wherein HeLa cells expressing the triple mutant showed an absence of both cytosolic and nuclear P-STAT1.

### U-STAT1 prevents the nuclear accumulation of co-expressed WT molecules in a concentration-dependent manner

**Fig. 2:**
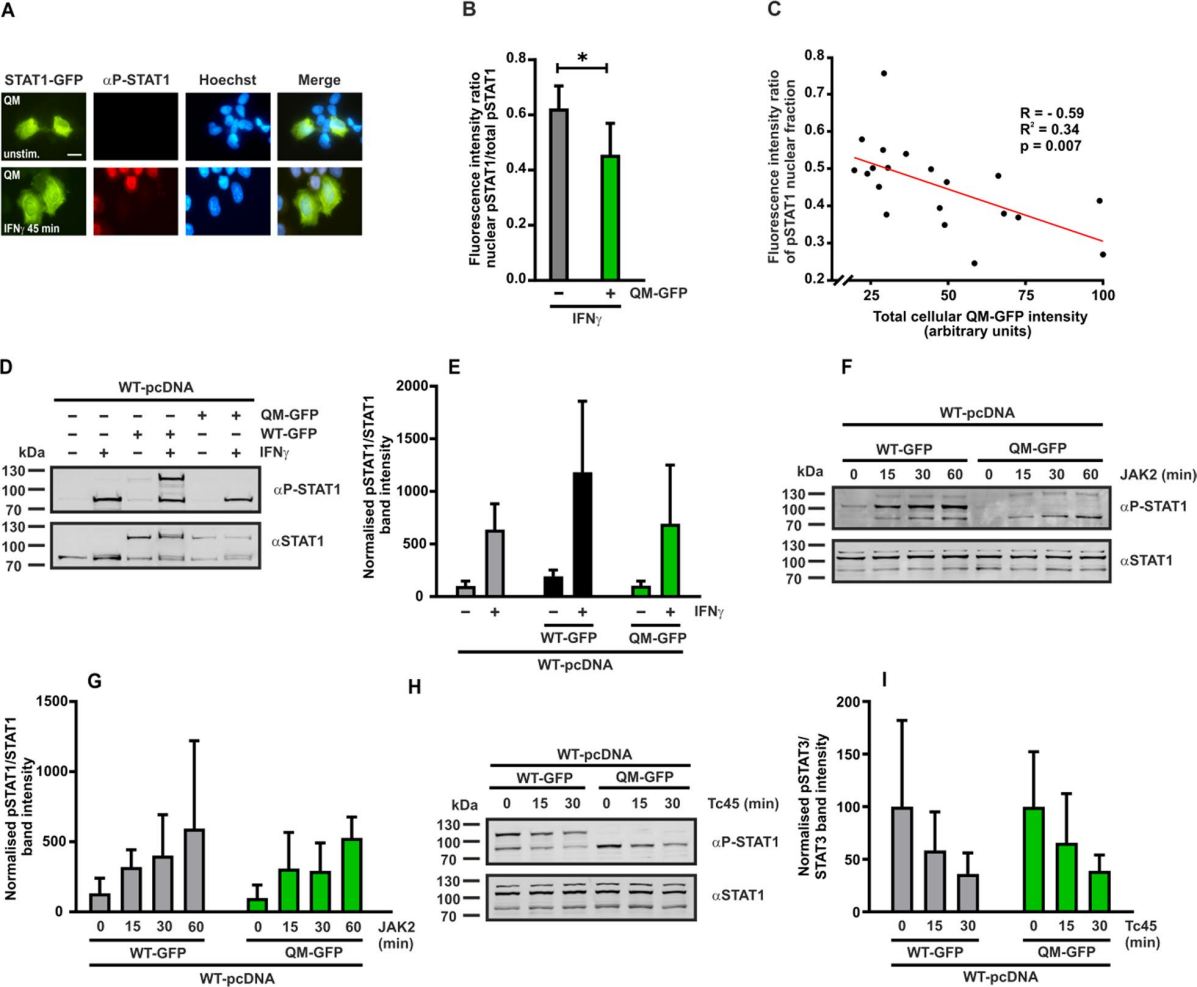
The dimerization-deficient quadruple mutant (QM-GFP) mimicking U-STAT1 inhibits the detection of co-expressed WT protein but does not affect tyrosine phosphorylation. **A**–**C** HeLa cells were transfected with a plasmid coding for QM-GFP and stimulated with IFNγ for 45 min followed by staining with anti-phospho-tyrosine antibody. **A** The fluorescence micrographs show the intracellular distribution of QM-GFP and WT P-STAT1, as well as the localization of the corresponding Hoechst-stained nuclei. **B** Histogram demonstrating the net reduction of nuclear P-STAT1 intensity in HeLa cells expressing QM-GFP, as determined by the ratio of nuclear-to-total fluorescence intensity (n = 3 independent transfections, means ± standard deviations from n = 20 cells, *p ≤ 0.05 by a two-tailed Student’s *t* test). **C** Concentration-dependent inhibition of nuclear P-STAT1 accumulation by co-expressed QM-GFP. The fluorescence intensities of nuclear P-STAT1 staining were plotted against the total cellular QM-GFP fluorescence. **D**, **E** Representative immunoblot of whole cell extracts from STAT1-negative U3A cells co-expressing untagged STAT1 and QM-GFP after treatment for 45 min with 50 ng/ml of recombinant IFNγ and the quantification thereof from three independent transfection experiments. **F**, **G** An in vitro phosphorylation assay demonstrated no difference in tyrosine phosphorylation rates of the WT STAT1 by JAK2 with respect to the presence or absence of QM-GFP. Whole cell extracts (10 μl in each reaction) from reconstituted U3A cells expressing untagged STAT1 in combination with WT-GFP or QM-GFP were incubated with 4 μg/ml of recombinant JAK2 kinase and the levels of P-STAT1 were monitored over time by means of Western blotting (n = 3). Statistical analysis revealed no significant difference in the phosphorylation kinetics of the WT in the presence of either QM-GFP or WT-GFP. **H**, **I** Results from an in vitro dephosphorylation assay using extracts from IFNγ-pre-stimulated U3A cells expressing untagged STAT1 in combination with WT-GFP or QM-GFP (10 μl each) incubated for 0, 15 and 30 min with 2 U of the STAT-specific Tc45 phosphatase (n = 3). Tyrosine dephosphorylation was followed by immunoblotting including a quantitative analysis of the phospho-tyrosine signals divided by total amount of STAT1 signal. Scale bar in **A** marks a distance of 10 µm

Previously, we had reported that the T385A mutation in the DNA-binding domain displayed a similar GOF phenotype as the R274W mutation with increased transcriptional activation due to reduced stability of the anti-parallel dimer [24]. In order to completely disrupt all known parallel and anti-parallel dimeric interactions in the core fragment of U-STAT1, we created a quadruple mutant (QM) containing four mutations R274W/T385A/R602L/Y701F, transfected this expression plasmid in HeLa cells and stimulated the cells for 45 min with IFNγ on the next day. These cytokine-stimulated cells expressing QM-GFP showed a significantly altered cellular distribution of P-STAT1, as seen from the almost complete absence of any positive immunoreactivity in the nuclei of the transfected cells stained with a phospho-tyrosine-specific antibody (Fig. 2A, B). Moreover, the intensity of the total cellular immunopositivity appeared to be significantly reduced, since tyrosine-phosphorylated endogenous STAT1 was hardly detectable by means of an antibody staining in cells expressing the QM-GFP, when compared to the bright staining in the adjacent, untransfected cells. Furthermore, the decrease in the observed P-STAT1 immunopositivity was dependent on the concentration of co-expressed QM-GFP molecules, as cells expressing a high number of QM-GFP proteins (bright green) showed a stronger reduction of detectable P-STAT1 as compared to cells expressing fewer mutant STAT1 molecules (dim green) (Fig. 2C).

Next, we performed Western blotting experiments in order to determine any changes in the tyrosine-phosphorylation levels of transfected WT STAT1 in samples co-expressing the QM protein. Results showed that, in whole cell extracts from IFNγ-stimulated STAT1-deficient U3A cells reconstituted with a combination of untagged WT STAT1 and QM-GFP, there was no reduction in the tyrosine phosphorylation of co-transfected WT STAT1. Despite the considerably reduced signal intensity in the fluorescence labelling observed in the micrographs using an anti-phospho-specific antibody, the additional presence of QM-GFP in these cells did not impair IFNγ-induced tyrosine phosphorylation of the WT protein in Western blot results (Fig. 2D, E). This unexpected finding can only be interpreted in a way that the tyrosine-phosphorylated WT STAT1 present in cells co-expressing QM-GFP cannot be detected using fluorescence staining, probably because the epitope is not accessible to the antibody, whereas it is detectable in denatured samples used for SDS-PAGE.

Extracts from either unstimulated or cytokine-stimulated U3A cells expressing a combination of WT and mutant STAT1 were used to perform in vitro phosphorylation and dephosphorylation assays, respectively, to investigate any interference of QM-GFP in the activation-inactivation cycle of co-expressed WT STAT1. For in vitro phosphorylation assays, extracts from unstimulated cells were incubated with the recombinant JAK2 enzyme, and for the dephosphorylation assays, extracts from cells pre-treated with cytokine were exposed to the recombinant STAT-inactivating Tc45 phosphatase. Both assays were performed for the indicated times, and subsequently probed using Western blotting experiments. The additional presence of QM-GFP expressed in these cells did not interfere with the kinetics of tyrosine phosphorylation and dephosphorylation of the co-expressed WT molecule. Untagged STAT1 phosphorylated (Fig. 2F, G) and dephosphorylated similarly, irrespective of the co-expression of either QM-GFP or WT-GFP (Fig. 2H, I). From these results, we inferred that a high concentration of dimerization-deficient, unphosphorylated STAT1 prevents nuclear accumulation of the co-expressed WT protein but does not alter its kinetics of tyrosine phosphorylation.

### Co-expressed WT shows unaltered DNA binding and transcriptional response in the presence of dimerization-deficient, unphosphorylated STAT1

**Fig. 3:**
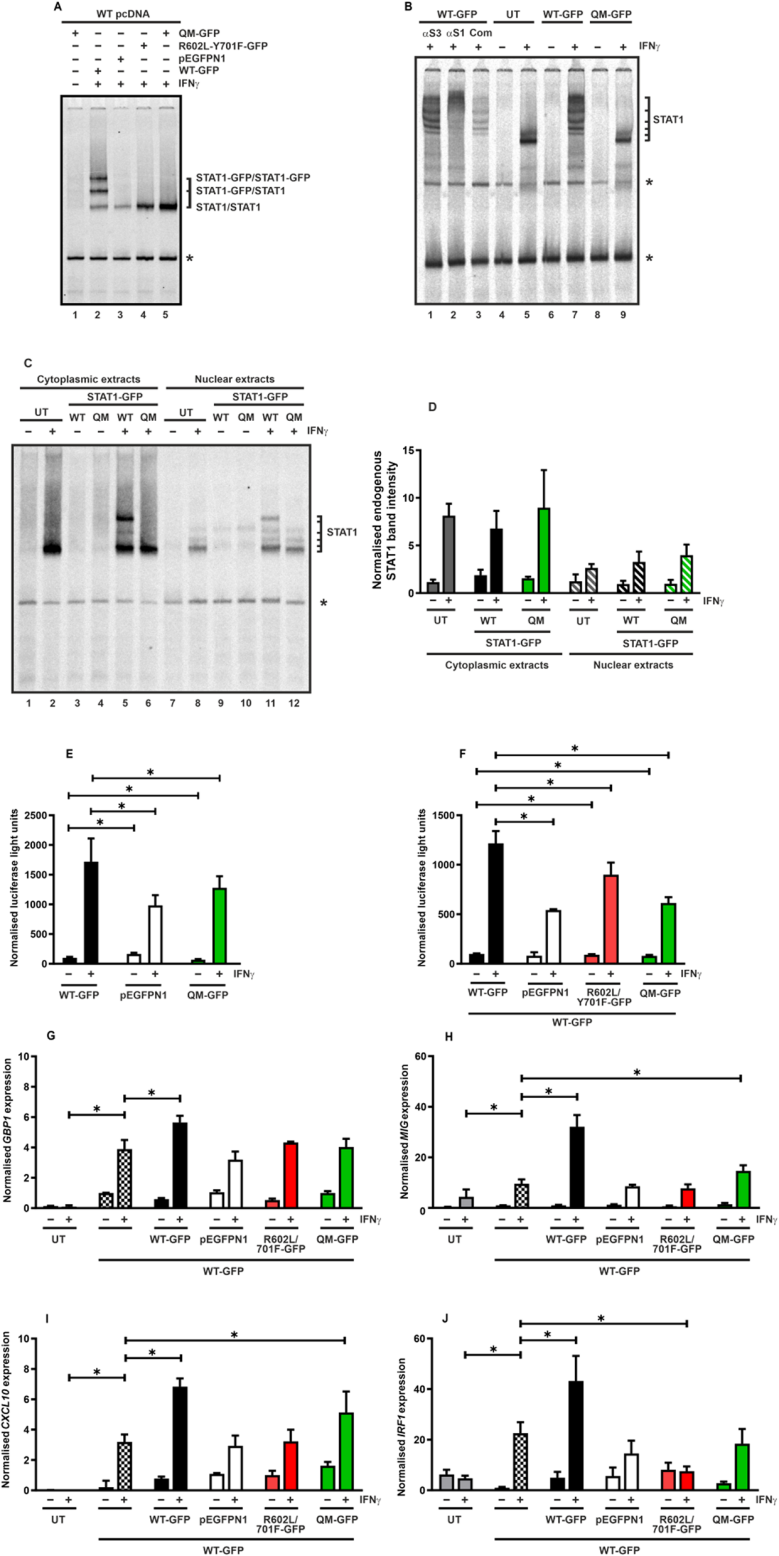
Unaltered DNA binding, reporter activation and target gene induction by WT STAT1 in the presence of a dimerization-deficient mutant. **A** Electrophoretic mobility shift assay demonstrated unchanged binding of the recombinant WT protein from reconstituted U3A cell extracts to a [^33^P]-radioactively labelled M67 probe containing a single GAS sequence, whereas the co-expressed unphosphorylated STAT1 variants and empty vector (pEGFPN1) showed no DNA-binding indicated by the absence of a GFP-tagged STAT1 band in lanes 3 to 5. The asterisk at the margin of the gelshift indicates an unspecific band. **B** EMSA result showing the unaltered capacity of endogenous STAT1 from HeLa cell extracts to form tetramers on the radioactively-labelled DNA probe 2xGAS, containing two GAS sites in tandem orientation, in the presence of co-expressed QM-GFP. HeLa cells were either untransfected (UT) or transfected with the indicated expression plasmids, and on the next day the cells were treated for 45 min with IFNγ or left untreated. Extracts were incubated with 2xGAS DNA probed for 15 min before subsequently being challenged by a 750-fold molar excess of unlabelled M67 DNA incubated for 10 min at RT in a competition assay (comp). For super-shift assays, 20 ng of STAT1- (αS1) or STAT3-specific (αS3) antibody were added to the reaction for 40 min at RT. **C**, **D** EMSA result showing the unaltered cytoplasmic and nuclear fractions of endogenous P-STAT1 from HeLa cell lysates bound to a [^33^P]-radioactively labelled M67 probe containing a single GAS sequence, as the additional presence of QM-GFP did not hamper nuclear import of the endogenous STAT1 but its nuclear retention. **E** HeLa cells expressing WT-GFP, empty vector (pEGFPN1) or the quadruple mutant QM-GFP were left untreated (−) or stimulated for 6 h with 50 ng/ml of IFNγ (+). In whole extracts from these cells, luciferase luminescence of a reporter construct with a triple GAS site (3xLy6E) and the enzymatic activity of the co-expressed β-galactosidase were measured and represented graphically. **F** U3A cells were transfected with a STAT1-responsive luciferase reporter construct, a β-galactosidase expression vector and a combination of equal amounts of WT-GFP and the indicated STAT1 variants. These cells were either unstimulated (−) or stimulated with 50 ng/ml of IFNγ (+) and subsequently luciferase activity, normalized to the β-galactosidase expression, was measured in whole cell extracts and represented graphically. The experiment was repeated in six independent transfections at least three times. **G**–**J** U3A cells were untransfected (UT) or transfected with a plasmid coding for WT-GFP alone or a combination of WT-GFP and the indicated U-STAT1 variants or empty GFP vector (pEGFPN1). These cells where either untreated or stimulated with 50 ng/ml of IFNγ for 6 h. after which RNA was isolated and converted to cDNA. Histograms show the results from qPCR experiments for the following STAT1 target genes: **G** *GBP1*, **H** *MIG*, **I** *CXCL10* and **J** *IRF1*. Gene induction was normalized to the expression of the house-keeping gene *GAPDH*. Histograms show means and standard deviations wherein the significant differences between the IFNγ-stimulated variant samples and cells expressing a single transfection of WT protein are marked by bars and asterisks. The experiment was repeated three times

We then investigated the sequence-specific DNA-binding activity of the STAT1 variants and any impact of the mutant U-STAT1 on the transcriptional activity of the co-expressed WT. For gel-shift assays, STAT1-deficient U3A cells were co-transfected with a plasmid coding for untagged WT STAT1 and either WT-GFP, mutant U-STAT1 constructs or empty GFP vector. These cells were stimulated with IFNγ, before total protein was extracted, and the lysates were incubated with radioactively-labelled DNA probe M67 containing a single high-affinity GAS site. The reactions were separated by means of electrophoresis and whereas mutant U-STAT1 showed no GAS binding, the co-expressed WT bound to DNA irrespective of the presence of mutant U-STAT1 (Fig. 3A). The increased DNA-binding activity seen in lanes 4 and 5 most likely reflects the fact that cytoplasmic retention of P-STAT1 partially protects it from being dephosphorylated, as the STAT1-specific Tc45 phosphatase is predominantly located in the nucleus. To determine whether the mutant U-STAT1 interfered with the binding of co-expressed WT to GAS sites in tandem orientation, cellular lysates were isolated from STAT1-expressing, IFNγ-stimulated HeLa cells transfected with the STAT1 variants. The additional presence of mutant U-STAT1 in these cells did not hamper the binding of endogenous STAT1 to DNA probes containing two GAS sites (Fig. 3B). Super-shift reactions with antibodies against STAT1 as well as competition experiments with a 750-fold molar excess of unlabelled GAS elements confirmed the formation of tetrameric complexes comprising GFP-tagged and endogenous STAT1 in different stoichiometry. However, in extracts from cells expressing QM-GFP, tetrameric complexes of solely endogenous STAT1 were detected, indicating that the mutant U-STAT1 did not interfere with DNA binding of the WT protein. Notably, the co-expression of QM-GFP did not result in a reduction of GAS-bound WT STAT1, as opposed to the decreased nuclear presence of the WT protein seen in immunocytochemical stainings (Fig. 1C).

We additionally performed subcellular fractionation experiments with IFNγ-stimulated HeLa cells transfected with either WT-GFP or QM-GFP plasmids and used the separated cytoplasmic and nuclear lysates to assess DNA binding through gelshift experiments. As seen in Fig. 3C, D, endogenous P-STAT1 was detected in the nuclear compartment of HeLa cells co-expressing QM-GFP, indicating unaltered binding to GAS sites on DNA.

To study cytokine-induced transcriptional responses of the co-expressed WT in the presence of mutant U-STAT1, we performed reporter gene assays using either HeLa cells transfected with STAT1 variants (Fig. 3E) or STAT1-deficient U3A cells, which were transfected with a combination of both WT-GFP and STAT1 variants (Fig. 3F). The cells were additionally transfected with a luciferase reporter construct containing three GAS sites upstream of the luciferase gene and a β-galactosidase plasmid for normalization of transfection efficiency [21]. Upon stimulation of these cells with IFNγ for 6 h, luciferase reporter activity was measured and normalized by the corresponding β-galactosidase activity. While cells expressing a double amount (250 ng) of WT STAT1 plasmid showed highest reporter activity, replacing one half of this amount with mutant U-STAT1 plasmids (125 ng WT STAT1 plasmid mixed with 125 ng of U-STAT1 variant plasmids) showed reporter activities similar to that of cells expressing only half the amount of WT STAT1 plasmid (125 ng) (Fig. 3F). This indicated that the additional presence of U-STAT1 did not affect the transcriptional activation mediated by the co-expressed WT (Fig. 3E, F).

The impact of a high concentration of dimerization-deficient U-STAT1 was subsequently tested on the IFNγ-induced transcription of endogenous STAT1 target genes using real-time PCR. STAT1-deficient U3A cells were either left untransfected or transfected with a plasmid coding for WT-GFP. The cells transfected with the WT-GFP plasmid were either left alone or additionally co-transfected with equal amounts of vectors coding for GFP fusions of WT, R602L/Y701F or QM. Cells transfected with a combination of WT-GFP plasmid and empty vector served as a control. The induction of four STAT1 target genes namely *GBP1*, *MIG*, *CXCL10* and *IRF1* were tested after stimulation with IFNγ for 6 h. Similar to the reporter gene experiments, cells expressing twice the amount of WT-GFP showed the highest activation of STAT1 target genes, while the cells which expressed half the amount of WT-GFP or additionally co-expressed U-STAT1 variants showed similar induction patterns (Fig. 3G–J). Although we observed an impaired nuclear accumulation of P-STAT1 in the presence of high concentrations of U-STAT1 (Fig. 1), this did not result in a significant decrease in transcriptional activity.

### Dimerization-deficient U-STAT1 blocks nuclear accumulation of co-expressed WT through amino-terminal domain-mediated interactions

To determine the nature of the interactions between the co-expressed U-STAT1 and the phosphorylated WT molecule, which resulted in its hampered nuclear accumulation, several approaches were investigated. Firstly, we tested the role of DNA binding in the observed inhibitory effect of U-STAT1 on the nuclear accumulation of P-STAT1. To this end, two point-mutations, namely V426D/T427D, which have been previously characterised as DNA^minus^ for markedly reducing affinity to DNA, were introduced in the R602L/Y701F-GFP construct [25]. This R602L/Y701F/DNA^minus^-GFP, when expressed in HeLa cells and stimulated with IFNγ, was unable to restore nuclear presence of the endogenous P-STAT1 proteins, thereby, disproving the hypothesis that high concentrations of U-STAT1 in these cells inhibited the retention of P-STAT1 on DNA (Additional file 1: Fig. S1A).

The asymmetric unit of the crystal structure of STAT3 studied by Ren and colleagues contains a parallel dimer comprising two unphosphorylated STAT3 proteins, significantly different from the parallel dimer of STAT1 [26]. The binding interface within this parallel dimer is localized around the linker domain of STAT3, which renders an acute and narrow alignment of the two protomers, as opposed to the canonical STAT1 dimer [5, 26]. We were interested in exploring the contribution of this binding interface in stabilizing the interaction between P-STAT1 and U-STAT1, and, therefore, introduced two mutations at STAT1-homologous positions (E524A and R586E in R602L/Y701F-GFP) of the STAT3 residues involved in this binding interface from the asymmetric crystal unit [26]. Again, there was an absence of endogenous P-STAT1 from the nuclei of IFNγ-stimulated HeLa cells expressing the linker domain mutant E524A/R586E/R602L/Y701F-GFP (Additional file 1: Fig. S1A, B). We concluded these residues did not contribute to the interactions between dimerization-deficient U-STAT1 and co-expressed P-STAT1 and, hence, are not responsible for altering the cytokine-induced redistribution of P-STAT1.

The amino-terminal domain of STAT1 has been widely studied and found to play an important role in the oligomerization of STAT1 and in forming stable tetramers on adjacent GAS sites on DNA [6, 10, 27]. To test whether amino-terminal dimerization confers stability to a complex between mutant U-STAT1 and co-expressed P-STAT1, the two residues W37 and F77 contributing to dimer formation between two amino-termini were mutated in QM-GFP [5, 28]. Again, the two amino-terminal mutant constructs W37F/R274W/T385A/R602L/Y701F-GFP and F77A/R274W/T385A/R602L/Y701F-GFP displayed a similar inhibitory effect on the nuclear accumulation of co-expressed, endogenous P-STAT1 (data not shown).

**Fig. 4:**
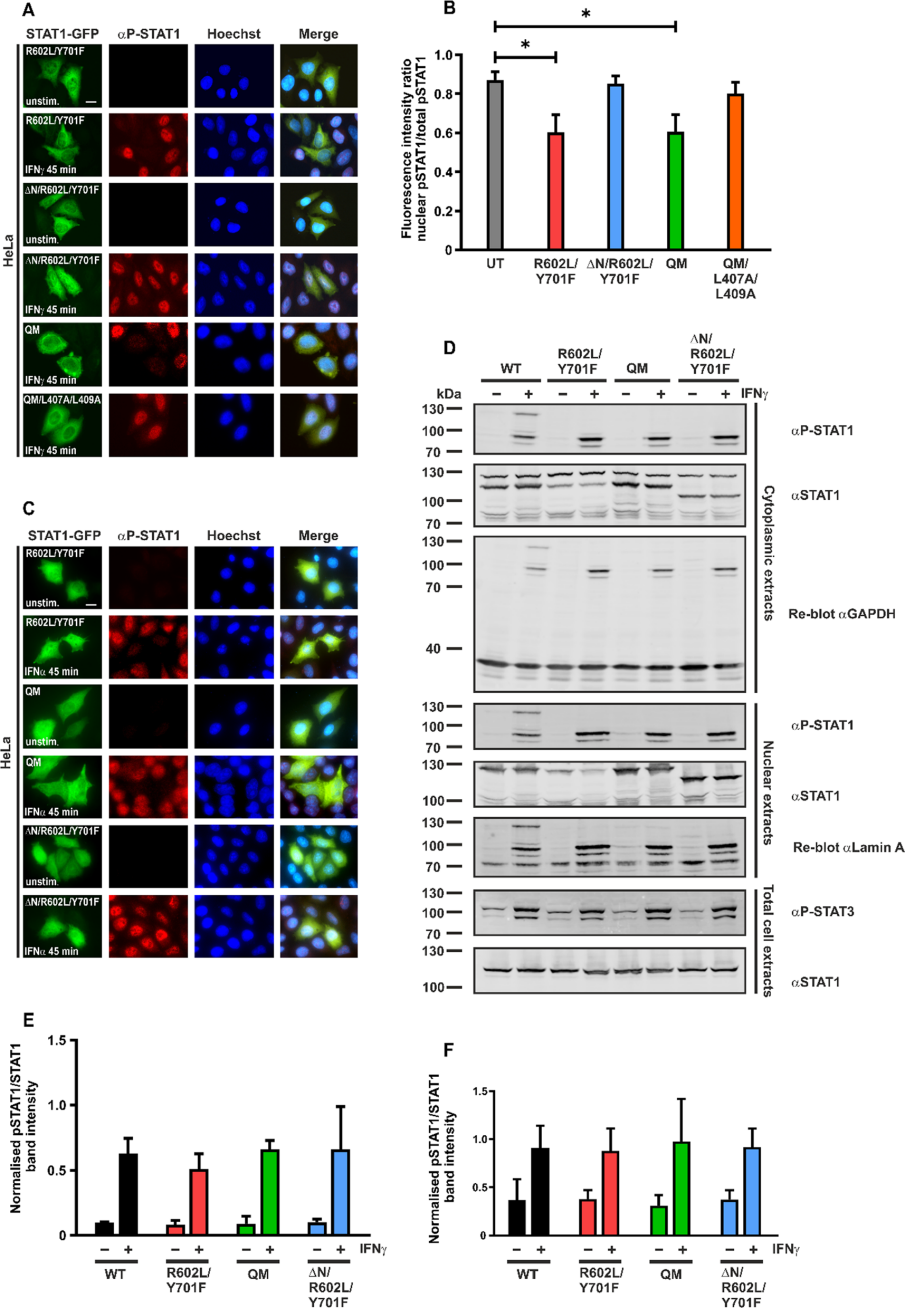
Deletion of the amino-terminus in mutant U-STAT1 restores nuclear accumulation of co-expressed, endogenous P-STAT1. **A**, **B** HeLa cells were transfected with an expression plasmid coding for R602L/Y701F-GFP, QM-GFP, ΔN/R602L/Y701F-GFP and QM/L407A/L409A-GFP and stimulated for 0 min or 45 min with 50 ng/ml of recombinant IFNγ, followed by staining with an anti-phospho-tyrosine antibody. Fluorescence micrographs show restored nuclear accumulation of P-STAT1 in the presence of amino-terminal deletion mutant ΔN/R602L/Y701F-GFP and the dsNLS mutant QM/L407A/L409A-GFP. Histograms show the quantification of nuclear P-STAT1 from HeLa cells co-expressing the indicated STAT1 variants in comparison with untransfected HeLa cells from three independent experiments (n = 3, means ± standard deviations from n = 20 cells, *p ≤ 0.05). **C** Fluorescence micrographs displaying the absence of nuclear endogenous P-STAT1 staining in the presence of mutant U-STAT1 constructs expressed in HeLa cells stimulated for 0 min and 45 min with recombinant IFNα (50 ng/ml). Note the rescue effect mediated by the deletion of the amino-terminus in the construct ΔN/R602L/Y701F-GFP under stimulation with type I IFN. Scale bars in **A**, **C** mark a distance of 10 µm. **D** Cellular fractionation experiments using cytoplasmic and nuclear lysates from HeLa cells expressing the indicated STAT1 variants demonstrate the presence of endogenous phospho-STAT1 in both compartments in IFNγ-pretreated cells. Representative immunoblots demonstrate unchanged tyrosine phosphorylation of endogenous STAT3 in whole cell extracts from HeLa cells expressing U-STAT1, after stimulation with IFNγ (lower panels). **E**, **F** Quantification from immunoblots of whole extracts from HeLa cells expressing the indicated mutants, including the quadruple mutant (QM), before and after treatment for 45 min with 50 ng/ml of recombinant IFNγ from three independent transfection experiments for the expression of phospho-STAT1 (**E**) and the expression of phospho-STAT3 (**F**)

In an alternative approach, the deletion of the amino-terminus in dimerization-deficient U-STAT1 was studied by introducing mutations R602L and Y701F in an expression vector coding for STAT1-GFP with an amino-terminal deletion. This generated a ΔN/R602L/Y701F-GFP construct which was transfected in HeLa cells and the cells were subsequently stimulated with IFNγ. Notably, cells expressing the construct comprising the double mutation R602L/Y701F along with the amino-terminal deletion completely rescued the disrupted nuclear accumulation of P-STAT1 (Fig. 4A, B; Additional file 1: Fig. S1C). The nuclei of IFNγ-stimulated HeLa cells expressing ΔN/R602L/Y701F-GFP showed a clear accumulation of endogenous P-STAT1, which was comparable to the neighbouring untransfected cells. The ΔN/R602L/Y701F-GFP mutation did not affect the phosphorylation status of the co-expressed WT protein in HeLa cells, as shown in Western blotting experiments (Fig. 4D, E). We also detected P-STAT1 in nuclear lysates from IFNγ-stimulated HeLa cells transfected with the STAT1 variants in Western blotting experiments, indicating that the mutant U-STAT1 allowed nuclear import of the WT protein but inhibited its nuclear retention (Fig. 4A, E). In order to investigate the effect of U-STAT1 on the activation of endogenous STAT3, we performed Western blotting experiments on lysates from IFNγ-stimulated HeLa cells expressing U-STAT1 and probed them using a phospho-Tyr705-specific STAT3 antibody. As shown in Fig. 4D, F, the co-expression of STAT1 variants had no significant impact on tyrosine phosphorylation of endogenous STAT3.

In summary, the amino-terminal deletion completely restored the nuclear accumulation and detection of P-STAT1, which was hampered by the presence of the double mutant. Similar results were obtained in immunocytochemistry experiments with HeLa cells stimulated with IFNα, which is known to exhibit strong antiviral effects through stimulated Janus kinase activity [29]. In IFNα-stimulated cells, expression of R602L/Y701F-GFP and QM-GFP inhibited nuclear accumulation of endogenous STAT1, but the nuclear presence of P-STAT1 in cells expressing ΔN/R602L/Y701F-GFP was similar to that of untransfected cells (Fig. 4C).

In order to study the molecular organization of the import complex comprising tyrosine-phosphorylated STAT1, a dimer-specific nuclear localization signal (dsNLS) located in a conserved motif consisting of residues L407 and L409 in the DNA-binding domain was mutated [14]. This sequence has been shown to be specific for the STAT1 dimer and when disrupted, STAT1 failed to undergo nuclear translocation upon activation and did not induce subsequent gene transcription in spite of normal DNA-binding capability [14]. Recognition of the STAT1 dsNLS by importin α is a prerequisite for the assembly of an import complex upon cytokine stimulation and we hypothesized that a disruption of this motif in QM/L407A/L409A-GFP would also restore nuclear P-STAT1 which had previously been lost in the presence of QM-GFP. Similar to ΔN/R602L/Y701F-GFP, the construct QM/L407A/L409A-GFP also completely reversed the lack of detectable nuclear accumulation of P-STAT1 observed for QM-GFP (Fig. 4A, B). These findings implied that the cytokine-induced import complex of STAT1 requires dsNLS recognition and additional interactions between the amino-terminal domains of the STAT1 cargo and the importins.

## Discussion

In the present study, we investigated the role of unphosphorylated STAT1 on the localization and signal transduction of its phosphorylated form. For this purpose, the double mutant R602L/Y701F was created such that this variant remains constitutively unphosphorylated, thereby mimicking U-STAT1. Due to its inability to form parallel dimers through reciprocal phosphotyrosine-SH2 interactions owing to a lack of the phosphorylatable tyrosine residue at position 701, it was expected that this construct would not have any effect on the intracellular shuttling of the co-expressed WT protein. However, in both HeLa and U3A cells expressing R602L/Y701F-GFP, the co-expressed WT molecule did not accumulate in the nucleus upon cytokine stimulation. Moreover, upon immunofluorescence staining with a P-STAT1 antibody, endogenous tyrosine-phosphorylated STAT1 could not be detected in either the cytosol or nuclei of IFNγ-stimulated HeLa cells expressing R602L/Y701F-GFP (Fig. 1). This inhibitory effect on the detection of P-STAT1 was also observed in IFNα-stimulated cells expressing R602L/Y701F-GFP, indicating that the lack of immunopositivity for the phosphorylated WT protein caused by this co-expressed mutant was independent of the type of cytokine used for stimulation (Fig. 4C).

To disrupt known dimerization interfaces, we introduced the additional mutations R274W and T385A in a step-wise manner in R602L/Y701F-GFP, to render a quadruple mutant, QM-GFP, which cannot form parallel or antiparallel dimers with P-STAT1. However, both R274W/R602L/Y701F-GFP and QM-GFP similarly inhibited nuclear accumulation of endogenous P-STAT1. A note-worthy phenomenon was the observed concentration-dependent nature of the inhibitory effect of QM-GFP on endogenous nuclear P-STAT1. In cells expressing a high level of the dimerization-deficient mutant, there was a greater reduction in nuclear P-STAT1, whereas a low concentration of mutant U-STAT1 inhibited the detection of nuclear P-STAT1 to a lesser degree.

Western blotting experiments in U3A cells expressing a combination of both recombinant WT and mutant protein or, alternatively, HeLa cells expressing native STAT1 and mutant U-STAT1 showed unchanged tyrosine phosphorylation of the WT protein. The tyrosine phosphorylation of STAT3 remained unaffected by the co-expression of mutant U-STAT1. In addition, unchanged kinetics of the WT protein in in vitro phosphorylation and dephosphorylation assays demonstrated that the activation/inactivation of WT STAT1 was unaltered in the presence of large amounts of mutant U-STAT1 though, nonetheless, cytokine-stimulated nuclear accumulation of WT protein could not be detected. While the U-STAT1 variants did not bind to DNA or induce transcription on their own, their additional presence neither altered WT-mediated GAS binding nor IFNγ-induced activation of STAT1 target genes. Despite the almost complete U-STAT1-mediated inhibition of the nuclear accumulation of P-STAT1, no gross changes occurred in the downstream signaling activities of P-STAT1 upon over-expression of U-STAT1.

To resolve the molecular mechanism of the U-STAT1-derived disruption of P-STAT1 nuclear accumulation, the V426D/T427D substitutions resulting in a decreased STAT1 affinity for DNA as well as the E524A/R586E substitutions in the linker domain were studied in combination with the dimerization-deficient mutations. Neither of these additional mutations restored the absence of nuclear P-STAT1 in cells stimulated with IFNγ. These results ruled out the possibility that high numbers of mutant U-STAT1 molecules could displace DNA-bound P-STAT1 through competition for binding sites, thereby, reducing nuclear retention. Furthermore, mutations in the linker domain of U-STAT1 did not reverse the inhibition of the nuclear accumulation of co-expressed WT protein.

It is known that two adjacent dimers bridge on DNA through reciprocal interactions between their amino-terminal domains, thereby stabilizing tetrameric STAT1 complexes and facilitating cooperative DNA binding [28, 30]. To study any possible contribution of the amino-terminus to the observed association between U-STAT1 and P-STAT1, this domain was deleted in the dimerization-deficient mutant R602L/Y701F-GFP. In contrast to the previous mutants, the generated ΔN/R602L/Y701F-GFP construct showed a distinct accumulation of P-STAT1 in the nuclei of IFNγ-stimulated cells. Thus, the absence of P-STAT1 nuclear retention induced by a high concentration of mutant U-STAT1 was relieved upon disruption of amino-terminal interactions in this variant by the complete deletion of the amino-terminal domain. Furthermore, a disruption of the dimer-specific NLS by two additional mutations L407A and L409A in QM-GFP also relieved the inhibition of P-STAT1 nuclear accumulation that had previously been seen in HeLa cells expressing QM-GFP. This indicates that both amino-terminal interactions and NLS binding are essential for the assembly of the STAT1-importin-α complex, which undergoes cytokine-induced import and subsequent retention in the nucleus.

The amino-terminal domain of STAT1 is required for associations with importin α5 upon stimulation with IFNγ, as the ΔN mutant of STAT1-GFP does not accumulate in the nucleus upon cytokine stimulation [31]. Therefore, P-STAT1 molecules bound to importins via NLS recognition could be possibly stabilized by additional interactions with their amino-terminal domain. Based on our data, we suggest that high levels of U-STAT1 interfere with the assembly of P-STAT1 into an importable complex which impedes its nuclear accumulation and inhibits the detection of tyrosine-phosphorylated STAT1 using immunofluorescence staining.

**Fig. 5:**
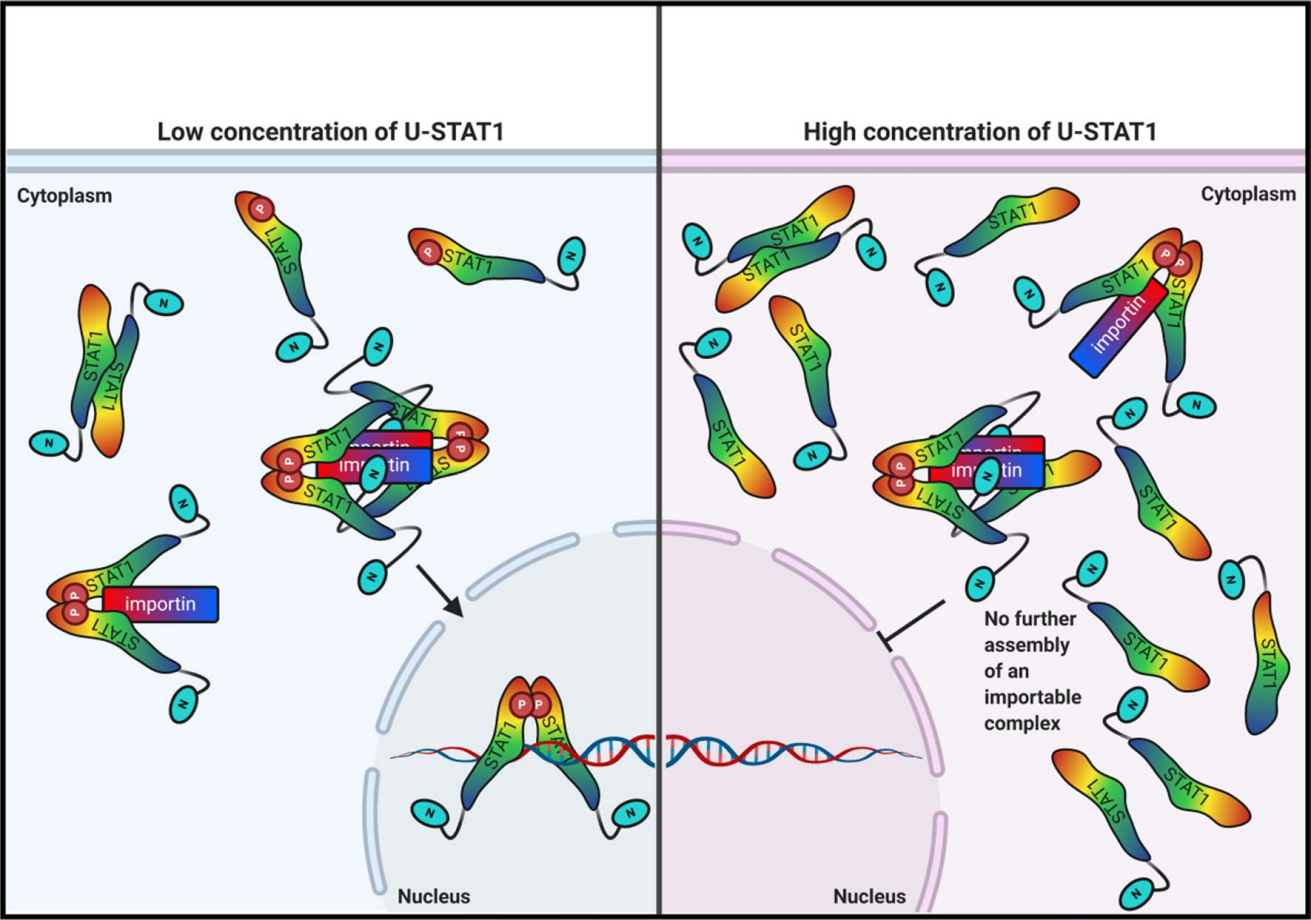
Proposed model of U-STAT1-dependent import modulation of P-STAT1. The left panel describes a condition of low-level cellular U-STAT1, wherein fully functional import complexes of P-STAT1 are formed and translocated to the nucleus in cytokine-stimulated cells. The right panel depicts a high concentration of U-STAT1, wherein U-STAT1 interacts through its amino-terminus and dsNLS with both components of the importin dimer. The formation for a full-fledged import complex is hindered by the missing tyrosine phosphorylation residue of U-STAT1

In this paper, we propose a model illustrating the effects of U-STAT1 on the nucleocytoplasmic translocation of P-STAT1, as shown in Fig. 5. Under low levels of STAT1 (left panel), cytokine-induced tyrosine phosphorylation leads to the formation of P-STAT1 parallel dimers, which bind to importins and are translocated to the nucleus. In the presence of low concentrations of U-STAT1, most of the P-STAT1 proteins form the importable complex and are rapidly translocated to the nucleus upon cytokine stimulation, facing little resistance from the low concentration of U-STAT1 in the cell. While Nardozzi and colleagues suggested that P-STAT1 binds to importin in a 2:1 stoichiometry [32], additional reports suggest that a rapid translocation of larger cargoes requires more than one transport molecule for import [33]. In addition, it is known from crystallographic data that the nuclear import factor karyopherin α, also known as importin α, forms a dimer [34]. Based on this, we hypothesized a higher-order importable complex comprising a dimer of importins bound to two P-STAT1 dimers additionally stabilized via STAT1 amino-terminal interactions, as illustrated in the left panel of Fig. 5. The assembly of P-STAT1 molecules into importable structures will be impeded by a high number of U-STAT1 proteins. In this scenario, U-STAT1 interacts through its amino-terminus and dsNLS with the importin-bound P-STAT1 dimer, thereby halting further assembly into a fully importable complex (Fig. 5, right panel). This will result in a cytoplasmic retention of P-STAT1, as observed in our experiments.

However, the nuclear translocation of P-STAT1 is not completely inhibited in the presence of mutant U-STAT1, as we observed unaltered transcriptional activity of the co-expressed WT protein in gene expression experiments and detected the phospho-protein in fractionation assays. Tyrosine-phosphorylated STAT molecules utilize tetramerization and cooperative DNA binding to initiate gene transcription which minimizes the need for all the P-STAT1 proteins in the cell to undergo nuclear import [27, 35]. The nuclear entry of a limited fraction of P-STAT1 molecules suffices to drive the transcription of cytokine-regulated genes [9]. Western blotting of denatured extracts allows the detection of these small amounts of phospho-protein from specific subcellular compartments, as could be seen from the presence of P-STAT1 in nuclear extracts. However, the detection of native multimeric protein structures within cellular compartments by means of a phosphotyrosine-specific antibody is highly sensitive to changes in the concentration of target proteins or conformational rearrangements that may mask the epitope. Since the mutations in R602L/Y701F-GFP and QM-GFP rule out direct interactions with P-STAT1 that may mask its phosphotyrosine epitope, a large number of mutant U-STAT1 proteins mostly likely interferes with the formation of multimeric P-STAT1 import structures and their subsequent antibody-based detection. Therefore, from our model we hypothesize that the high levels of cytoplasmic U-STAT1 molecules may have a function in regulating the compartmentalization of P-STAT1 upon cytokine stimulation, with little effect on its downstream signal transduction.

Several other studies have reported different effects of U-STAT1 on IFN I and II signalling pathway. Mice carrying the heterozygous Y701F mutation showed attenuated IFN-α responses due to an inhibition of the nuclear accumulation of STAT2 and a resulting compromised immunity against *Legionella pneumophila* [36]. The lack of observed nuclear STAT2 in murine bone-marrow derived macrophages in the presence of the Y701F mutant hints at an association between the unphosphorylated and phosphorylated STAT protein with the potential to alter the cellular distribution of the phospho-protein. Furthermore, semi-phosphorylated heterodimers comprising unphosphorylated STAT2 and phosphorylated STAT1 have been previously reported to be incapable of binding to importin-α and subsequently retaining activated STAT1 in the cytosol [37]. However, the impact of U-STAT1 on the nucleo-cytoplasmic distribution of P-STAT1 has not been investigated before, which we have now examined in this paper. The findings from the studies discussed above introduce an inhibitory role of the unphosphorylated STAT protein on the IFN response mediated by P-STAT1. The observations reported in our study differ in this respect, as we introduce a modulatory role of U-STAT1 on the intracellular distribution of P-STAT1.

Our proposed import model is corroborated by reports underlining the significance of the amino-terminal domain in the dimerization of other unphosphorylated STAT proteins and their nuclear accumulation upon cytokine stimulation. The Müller-Newen laboratory showed that a deletion of the amino-terminal domain precluded the dimerization of unphosphorylated STAT3 and its nuclear accumulation upon IL-6 exposure [38]. The STAT3 amino-terminal domain has also been implicated in exerting the dominant-negative activity of the Y705F mutant on the WT protein, by facilitating the formation of semi-phosphorylated dimers and preventing the homodimerization and subsequent nuclear accumulation of the WT [39]. We additionally propose here a role of the STAT1 amino-terminal domain in the formation of stable STAT1-importin α5 complexes, such that a deletion of this domain in mutant U-STAT1 fully restored P-STAT1 nuclear accumulation. As shown in Figs. 3 and 4, using subcellular fractionation, we detected the presence of P-STAT1 in the nuclear compartment that drives the reporter activity and target gene induction observed in our experiments. Amino-terminal deletion studies in STAT1 and STAT3 both showed reduced nuclear accumulation of the ΔN constructs, and a faster nucleocytoplasmic shuttling kinetics of STAT3-ΔN as compared to the WT protein, which may explain its reduced nuclear retention [31, 38]. These observations corroborate the structure of our proposed tetrameric STAT1-importin complex. According to our model, the formation of the STAT1-importin α5 complex requires the expression of a STAT1 protein with an intact amino-terminus.

Based on our data, we suggest a constitutive action of unphosphorylated STAT proteins in regulating the redistribution of the STAT pool upon cytokine stimulation. The dissolution of STAT1 paracrystal formation by small-ubiquitin-like-modifier (SUMO) conjugation is an example of a similar inherent mechanism to regulate STAT1 activity and hyperresponsiveness to IFNγ [40, 41]. While SUMOylation directly inhibits the activity of STAT1, the U-STAT1 variants reported in this study only altered the nuclear aggregation of P-STAT1 without impacting signal transduction. This U-STAT1-mediated modulation of the nuclear import of P-STAT1 can be of relevance in the event of IFN priming on macrophages and monocytes, wherein the level of STAT1 is increased through a positive feedback loop as STAT1 upregulates its own expression [42–44]. The high concentration of newly synthesized U-STAT1 in IFN-primed cells is largely retained in the cytosolic compartment, where it can execute apoptotic functions [18]. Whereas nuclear localization of P-STAT1 is not required for the induction of caspase genes, it is the cytoplasmic presence of U-STAT1 that triggers the apoptotic machinery through the up-regulation of caspase gene expression [45, 46]. We hypothesize that in cells primed with IFNγ, the import modulation mediated by U-STAT1 ensures that the rate of STAT1 nuclear import is buffered, whereby the IFNγ-induced peak response of P-STAT1 nuclear build-up is flattened into a rather continuous, sustained release into the nucleus over time. This allows the transcriptional response mediated by P-STAT1 to be grossly unchanged as compared to IFN-treated, non-primed cells, while retaining the cellular susceptibility to apoptosis executed by cytoplasmic U-STAT1. This mechanism uncouples the cytoplasmic activities of STAT1 facilitating apoptosis from the unaltered cytokine-induced transcriptional activity in the nucleus. Retaining sensitivity to cytokines as well as the ability to induce apoptosis is crucial to the cell in the event of a microbial infection. Therefore, U-STAT1 functions as a double-edged sword, preserving full-fledged interferon signal transduction while directing pathogen-exposed cells to apoptosis.

## Conclusion

Using a mutational approach, we investigated the interactions between synthetic constructs that mimic unphosphorylated STAT1 (U-STAT1) upon cytokine stimulation of cells and revealed a unique role of U-STAT1 in inhibiting the nuclear accumulation of P-STAT1. The nuclear accumulation of P-STAT1 was diminished in the presence of mutant U-STAT1, which was not restored by mutations reducing the DNA affinity of mutant U-STAT1. Despite the lack of nuclear accumulation, significant amounts of STAT molecules can enter the nuclear compartment to induce cytokine-mediated gene expression. Based on these findings, we present a novel model wherein the amino-terminal domain in combination with the nuclear localisation signal of STAT1 are essential for the assembly of a STAT1 import complex, which regulates its nucleoctoplasmic distribution.

## Supplementary Information

**Additional file 1: Figure S1.** Reducing DNA binding affinity or mutations in the linker domain of mutant U-STAT1 does not restore the detection of co-expressed P-STAT1. (**A**) HeLa cells expressing DNA^minus^/R602L/Y701F-GFP with additional V426D/T427D mutations abolishing binding to DNA (DNA^minus^) or R602L/Y701F-GFP with two additional mutations E524A and R586E in the STAT1 linker domain, were either left untreated or stimulated with IFNγ followed by staining with an anti-phospho-tyrosine antibody. The fluorescence micrographs show the intracellular distribution of the U-STAT1 variants and endogenous P-STAT1, as well as the localization of the corresponding Hoechst-stained nuclei (n = 3 independent transfections). (**B**) Histogram demonstrating the net reduction in the intensity of nuclear P-STAT1 staining from HeLa cells expressing the corresponding mutants in comparison with adjacent untransfected cells, as determined by the ratio of nuclear-to-total fluorescence intensity (n = 3 independent transfections, means ± standard deviations from n = 20 cells, *p ≤ 0.05). (**C**) The lack of nuclear detection of endogenous P-STAT1 in cytokine-stimulated HeLa cells expressing R602L7Y701F-GFP, as assessed using laser scanning microscopy. HeLa cells expressing the indicated GFP-tagged STAT1 variants were treated for 45 min with IFNγ. Subsequently, the methanol-fixed and Hoechst-labelled cells were immunocytochemically stained using an anti-phospho-tyrosine Y701 antibody followed by a Cy3-conjugated secondary antibody. The subsequent rescue of this lack of nuclear accumulation can be seen upon deletion of the N-terminal domain in ΔN/R602L/Y701F-GFP (lower panel). Scale bars in (**A, C**) mark a distance of 10 µm.

## Data Availability

All data generated or analysed during this study are included in this published article and its Additional file 1.

## Abbreviations

STAT: Signal transducer and activator of transcription
JAK: Janus kinase
SH2: Src-homology 2
GFP: Green fluorescent protein
WT: Wild-type
P-STAT1: Phosphorylated STAT1
U-STAT1: Unphosphorylated STAT1
IFN: Interferon
NLS: Nuclear localization signal
GAS: Gamma-activated site
FBS: Fetal bovine serum
RPMI: Roswell Park Memorial Institute
SDS: Sodium dodecyl sulphate
HEPES: 4-(2-Hydroxyethyl)-1-piperazineethanesulfonic acid
PAGE: Polyacrylamide gel electrophoresis
EDTA: Ethylenediaminetetraacetic acid
DTT: Dithiothreitol
PBS: Phosphate buffered saline
RT: Room temperature
EMSA: Electrophoretic mobility shift assay
EGTA: Ethylene glycol-bis(β-aminoethyl ether)-N,N,N′,N′-tetraacetic acid
GAPDH: Glyceraldehyde 3-phosphate dehydrogenase
GOF: Gain-of-function
QM: Quadruple mutant
dsNLS: Dimer specific NLS
