# Supplementary material for: An inhibitory effect on the nuclear accumulation of phospho-STAT1 by its unphosphorylated form
Source: Cell Commun Signal. 2022 Mar 31;20:42. doi: 10.1186/s12964-022-00841-3 (PMC8974011; doi:10.1186/s12964-022-00841-3)

**Supplementary Figure:** Reducing DNA binding affinity or mutations in the linker domain of mutant U-STAT1 does not restore the detection of co-expressed P-STAT1. (**A**) HeLa cells expressing DNA^minus^/R602L/Y701F-GFP with additional V426D/T427D mutations abolishing binding to DNA (DNA^minus^) or R602L/Y701F-GFP with two additional mutations E524A and R586E in the STAT1 linker domain, were either left untreated or stimulated with IFNγ followed by staining with an anti-phospho-tyrosine antibody. The fluorescence micrographs show the intracellular distribution of the U-STAT1 variants and endogenous P-STAT1, as well as the localization of the corresponding Hoechst-stained nuclei (n=3 independent transfections). (**B**) Histogram demonstrating the net reduction in the intensity of nuclear P-STAT1 staining from HeLa cells expressing the corresponding mutants in comparison with adjacent untransfected cells, as determined by the ratio of nuclear-to-total fluorescence intensity (n=3 independent transfections, means ± standard deviations from n=20 cells, *p≤0.05). (**C**) The lack of nuclear detection of endogenous P-STAT1 in cytokine-stimulated HeLa cells expressing R602L7Y701F-GFP, as assessed using laser scanning microscopy. HeLa cells expressing the indicated GFP-tagged STAT1 variants were treated for 45 min with IFNγ. Subsequently, the methanol-fixed and Hoechst-labelled cells were immunocytochemically stained using an anti-phospho-tyrosine Y701 antibody followed by a Cy3-conjugated secondary antibody. The subsequent rescue of this lack of nuclear accumulation can be seen upon deletion of the N-terminal domain in ΔN/R602L/Y701F-GFP (lower panel). Scale bars in (**A, C**) mark a distance of 10 µm.


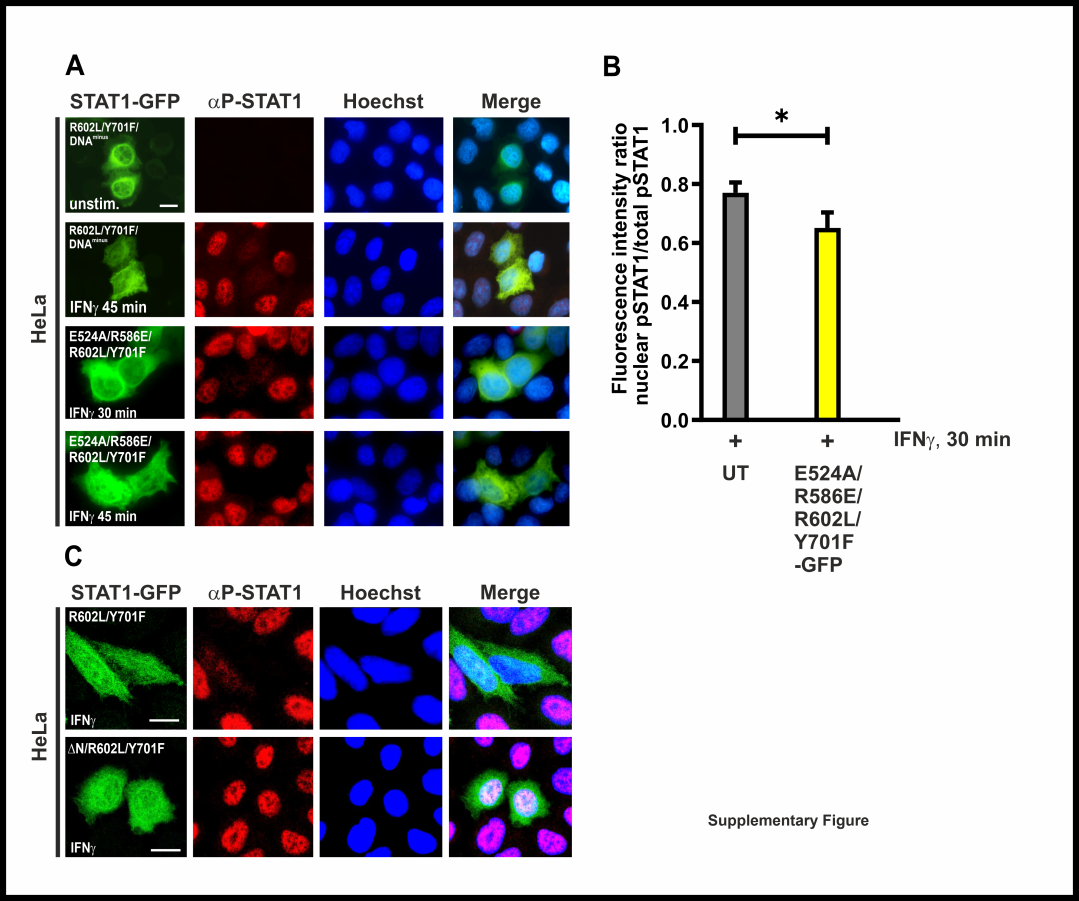

Supplement: Supplementary file 2 — Additional file 1: Figure S1. Reducing DNA binding affinity or mutations in the linker domain of mutant U-STAT1 does not restore the detection of co-expressed P-STAT1. (A) HeLa cells expressing DNAminus/R602L/Y701F-GFP with additional V426D/T427D mutations abolishing binding to DNA (DNAminus) or R602L/Y701F-GFP with two additional mutations E524A and R586E in the STAT1 linker domain, were either left untreated or stimulated with IFNγ followed by staining with an anti-phospho-tyrosine antibody. The fluorescence micrographs show the intracellular distribution of the U-STAT1 variants and endogenous P-STAT1, as well as the localization of the corresponding Hoechst-stained nuclei (n = 3 independent transfections). (B) Histogram demonstrating the net reduction in the intensity of nuclear P-STAT1 staining from HeLa cells expressing the corresponding mutants in comparison with adjacent untransfected cells, as determined by the ratio of nuclear-to-total fluorescence intensity (n = 3 independent transfections, means ± standard deviations from n = 20 cells, *p ≤ 0.05). (C) The lack of nuclear detection of endogenous P-STAT1 in cytokine-stimulated HeLa cells expressing R602L7Y701F-GFP, as assessed using laser scanning microscopy. HeLa cells expressing the indicated GFP-tagged STAT1 variants were treated for 45 min with IFNγ. Subsequently, the methanol-fixed and Hoechst-labelled cells were immunocytochemically stained using an anti-phospho-tyrosine Y701 antibody followed by a Cy3-conjugated secondary antibody. The subsequent rescue of this lack of nuclear accumulation can be seen upon deletion of the N-terminal domain in ΔN/R602L/Y701F-GFP (lower panel). Scale bars in (A, C) mark a distance of 10 µm. [file 12964_2022_841_MOESM2_ESM.docx]
